# Training of Field-Workers for Rapid Assessment of Scabies Prevalence: A Diagnostic Accuracy Study in Mozambique

**DOI:** 10.4269/ajtmh.24-0204

**Published:** 2024-09-18

**Authors:** Joanna Furnival-Adams, Valeria López, Hansel Mundaca, Amelia Houana, Antonio Macucha, Eldo Elobolobo, Aida Xerinda, Humberto Munguambe, Felisbela Materula, Regina Rabinovich, Francisco Saute, Daniel Engelman, Carlos Chaccour

**Affiliations:** ^1^ ISGlobal, Barcelona Institute for Global Health, Barcelona, Spain;; ^2^ Facultat de Medicina i Ciències de la Salut, Universitat de Barcelona (UB), Barcelona, Spain;; ^3^ Universidad Central de Venezuela, Caracas, Venezuela;; ^4^ Centro de Investigação em Saúde de Manhiça, Mopeia, Mozambique;; ^5^ Harvard T.H. Chan School of Public Health, Boston, Massachusetts;; ^6^ Tropical Diseases, Murdoch Children’s Research Institute, Melbourne, Australia;; ^7^ CIBERINFEC, Madrid, Spain;; ^8^ Instituto de Salud Tropical, Universidad de Navarra, Pamplona, Spain

## Abstract

Scabies is endemic in many resource-poor tropical areas, causing significant morbidity. However, our understanding of the true burden of scabies in Africa is limited, partly owing to limited capacity and challenges accessing the currently recommended diagnostic tools. The primary objective of this study was to assess the diagnostic accuracy of scabies assessments made by minimally trained field-workers. We trained field-workers with a minimum of secondary school education in the diagnosis of scabies. After the training, we assessed the diagnostic accuracy of assessments made by nine field-workers compared with the reference standard. In all, 193 individuals were assessed for scabies. The sensitivity, specificity, positive predictive value, and negative predictive value were calculated, as well as agreement (κ coefficients) between medical doctors and between field-workers. Of the 193 participants, 26% had scabies according to the reference standard. The sensitivity of field-worker diagnosis compared with the reference standard was 94% (95% CI: 90–99%), and the specificity was 96% (95% CI: 90–97%). The determination of severity by field-workers was less accurate; the sensitivity for severe scabies was 61% (95% CI: 48–74%), and the mean specificity was 97% (95% CI: 93–100%). This study demonstrated that field-workers without medical qualifications were capable of diagnosing scabies to a similar level of accuracy as experienced medical doctors after a short period of focal training. This may facilitate rapid assessments of scabies prevalence for public health purposes and decisions about mass drug administration implementation in similar settings.

## INTRODUCTION

Scabies is an ectoparasitic skin infestation, endemic in many tropical, resource-poor communities.^1^ Scabies mites cause skin lesions and intense itch that are often associated with secondary bacterial infections (impetigo), which can lead to more severe sequelae such as severe soft-tissue infection, rheumatic fever, post-streptococcal glomerulonephritis, and sepsis.^2^^,^^3^ In addition to the morbidity associated with scabies, it also has negative profound psychosocial and economic impacts that can perpetuate poverty. Furthermore, effective treatment can be difficult to access in rural, lower-middle income settings, meaning that episodes of scabies are often long-lasting and recurrent.

Our understanding of the true prevalence of scabies in the African region is extremely limited. A cross-sectional analysis of global scabies prevalence in 2017 reported no available data sources for sub-Saharan Africa (SSA) and relied on estimates from other continents and the covariate of water source to estimate the burden of scabies in this region.^4^ A more recent article reported estimates of scabies prevalence in eight African countries; however, the majority of these estimates were based on small-scale studies, and there were no available data from the remaining 46 countries.^1^ High-quality data collection has been impeded by the limited investment in scabies control programs and research. Paradoxically, high-quality data are needed to encourage investment into scabies disease programs and to generate data. Therefore, accessible and economic methods of assessing scabies prevalence are needed to further advocate for investment into scabies control programs.

In SSA, the most economically disadvantaged communities often live in rural, difficult-to-reach areas.^5^ Thus, individuals living with scabies often do not seek care; therefore, the number of cases seen at health facilities is not a reliable indicator of the true scabies burden in the community.^1^ Therefore, novel diagnostic approaches and tools for assessing scabies in field settings are needed.

Currently, there are no point-of-care diagnostic tests for scabies that are appropriate for use in field settings.^6^^,^^7^ Accessibility and availability of these tools would improve our ability to estimate population prevalence and provide information that could be used for control program decision-making. Scabies diagnosis in high-income clinical settings typically requires examination by an experienced doctor, and sometimes microscopic visualization of mites on skin scrapings is used to confirm diagnoses. However, these methods are not feasible in field settings, as they require highly skilled workers and specialized equipment. The need for improved diagnostic tools for scabies has been highlighted as a priority for global scabies control as part of the WHO 2030 road map for neglected tropical diseases.^8^^,^^9^ To accommodate this, consensus diagnostic criteria for scabies were developed in 2020 by a panel of experts convened by the International Alliance for the Control of Scabies (2020 IACS criteria), with the goal of promoting standardized case definitions of scabies and more comparable datasets.^10^^,^^11^ The 2020 IACS criteria define scabies cases as either confirmed, clinical, or suspected based on the level of diagnostic certainty.

Historically, medical doctors have been primarily responsible for diagnosing patients. However, task shifting, whereby tasks typically performed by a doctor are shifted to healthcare professionals with less-specialized positions, is now becoming more common.^12^ This can improve access to diagnostics and treatment in resource-poor areas. The same strategy may be used for scabies prevalence surveys. A few studies have assessed the accuracy of nonclinician healthcare professionals trained in the diagnosis of scabies in the field after the IACS criteria in the South Pacific and West African settings. One study evaluated the diagnostic accuracy of four briefly trained nurses who examined a cohort of 171 schoolchildren using clinical criteria.^13^ The researchers found that the nurses had high levels of sensitivity and specificity for moderate to severe scabies (94% and 74%, respectively) but low sensitivity for mild scabies compared with the consensus diagnosis of two expert physicians. In another study, two dermatologists trained five nurses and screened 135 people for scabies and found 55% sensitivity and 90% specificity, with again higher levels of sensitivity for moderate and severe cases.^14^ Finally, a study evaluated the use of a modified, simplified version of the IACS 2020 criteria for mapping and rapidly assessing scabies prevalence in very high–prevalence settings.^15^ This modified version had a sensitivity of 82% and a specificity of 98% compared with diagnosis using the full 2020 IACS criteria, with no difference in the pooled prevalence estimates using the two methods.

In 2022, a large-scale randomized controlled trial (BOHEMIA) took place in Mopeia, Mozambique, to assess ivermectin mass drug administration (MDA) (400 *µ*g/kg over 3 consecutive months) for malaria vector control and enrolled over 20,000 participants.^16^ A secondary objective of this clinical trial was to assess the impact of ivermectin MDA on the local prevalence of scabies. The field-workers who implemented the MDA had a minimum of a secondary school education to grade 10, and within this project, had received training related to the distribution of MDA and malaria screening. The resources to screen such a large population using medical doctors and microscopy were not available; therefore, we chose to train a subgroup of the same field-workers who implemented the MDA in the diagnosis of scabies. If nonmedical workers can accurately identify scabies cases, this may facilitate rapid assessments of scabies prevalence for public health purposes and decisions about MDA implementation in similar settings.

The primary objective of this study was to assess the accuracy of minimally trained field-workers’ diagnosis of scabies in terms of positive predictive value (PPV), negative predictive value (NPV), sensitivity, and specificity compared with diagnosis by experienced medical doctors.

## MATERIALS AND METHODS

### Study setting.

This study was nested within a trial that took place between March and September 2022 in Mopeia, Mozambique. This district is located in the central north of Mozambique and has a population of around 162,000.^17^ To our knowledge, to date there have been no large-scale published studies reporting scabies prevalence in Mozambique.^18^

### Training procedures.

We trained 90 individuals with high school qualifications over 2 days. Between February 4 and 7, 2022, 28 supervisors and 62 field-workers were trained in the diagnosis of scabies and infected scabies by J. Furnival-Adams, V. López, and A. Houana. J. Furnival-Adams has a background in infectious disease epidemiology and extensive knowledge of scabies pathology. V. López and A. Houana are medical doctors with experience working and diagnosing scabies in tropical, scabies-endemic settings. All field-workers had completed secondary education up to grade 10, but experience in healthcare settings was not a specified requirement. Field-workers were selected from a larger team of study workers based on observed performance in training for other trial activities. The training techniques were based on methods used in previous studies.^13^^,^^19^ Day 1 consisted of 5 hours of presentations and discussion on scabies and infected scabies. Day 2 included a written assessment (assessment 1) followed by a summary and discussion of the answers. The slides and structure of the training was designed by and agreed upon among the three trainers. The assessment included 50 photo-based case scenarios, where the trainees were required to assess the presence or absence of scabies lesions, quantify scabies lesions, and assess the presence or absence of impetigo. The trainees were encouraged to feel the skin using gloves to support their clinical assessments in the field. The ground truth for these photos was based on confirmed diagnoses by experienced medical doctors. A refresher training session took place (for field-workers only) on February 18, 2022, 11 days after the first sessions, and included a video demonstration, review of the scabies and impetigo presentations, and assessment practice using role-play. The field-workers then repeated the written assessment using the same case images in a different random order (assessment 2). Throughout the training sessions, the trainers asked the field-workers questions to reaffirm their knowledge, and they were encouraged to ask questions if anything was unclear. From these field-workers, a subsample of nine were randomly selected to take part in this substudy (this was the maximum number of field-workers that the project’s operational capacity allowed).

### Study design.

**Figure 1.**
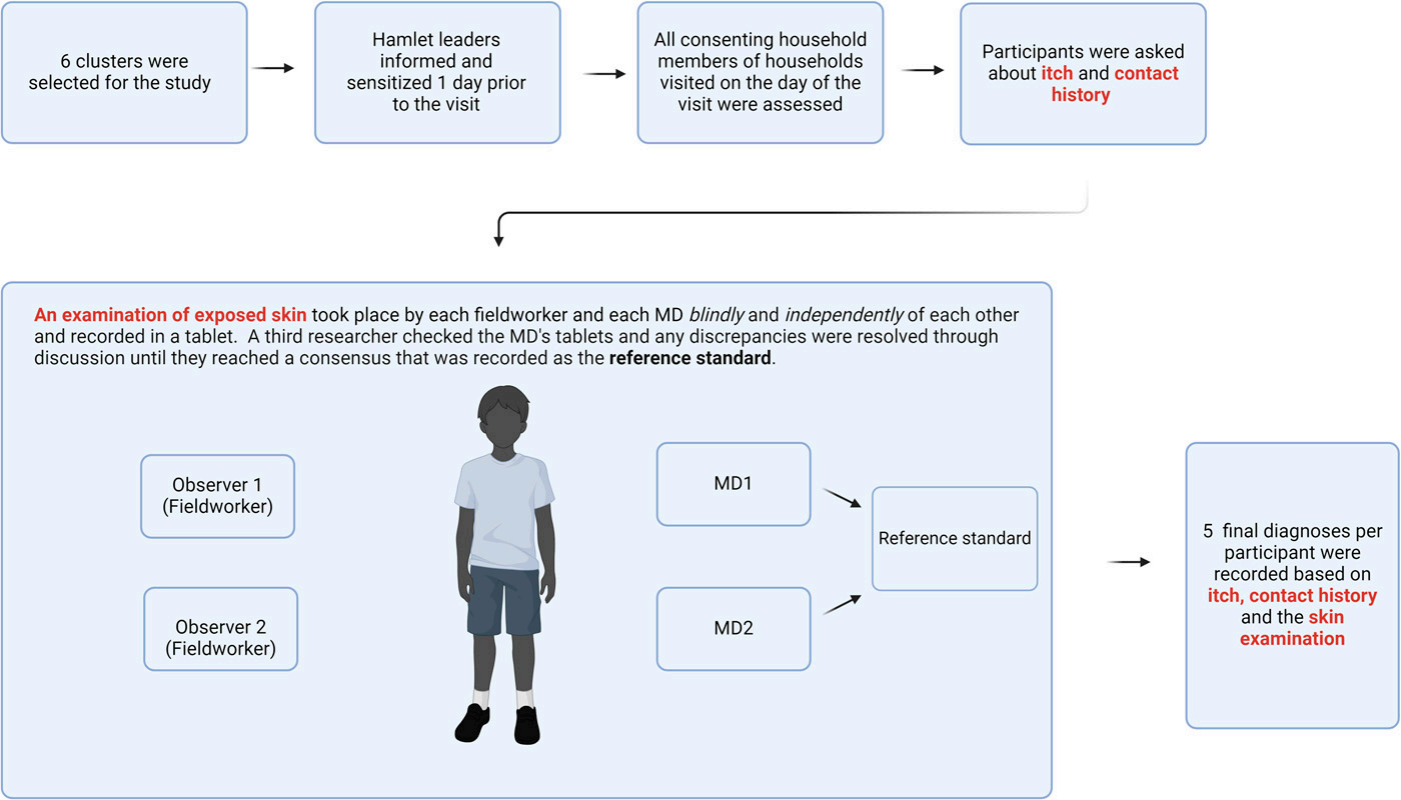
A schematic showing the sequence of events and processes involved in sampling, participant selection, and clinical assessments by the fieldworkers and medical doctors. MD = Medical Doctor.

A diagnostic accuracy study to assess the performance of the trained field-workers took place during a 3-week period in July 2022. Each day, two field-workers and the two medical doctors visited households that consented to participate in the study, within clusters that had previously reported high levels of body itch during the enumeration and census visits conducted in preparation for the trial (Figure 1).

The hamlet leaders of these communities were asked for verbal consent 1 day prior to our visit and made community members aware of the planned visit. All household members who were present on the day of the visit were screened. For every visit, two field-workers and the two medical doctors jointly asked participants questions about body itch within the past 24 hours and contact history (body itch among other household members within the past 24 hours). Field-workers rotated between each visit. For operational reasons, there was some variation in the number of visits per field-worker. Owing to the need for all assessors to understand the local languages, it was not possible to conduct this part of the diagnosis independently. After the questionnaire, the field-workers and medical doctors each independently examined the exposed skin of the participant. If lesions were present, following the IACS 2020 diagnostic criteria, the assessors counted the number of lesions, and this was used as an indicator of severity, with 1 or 2 lesions classified as very mild; 3–10 as mild; 11–49 as moderate, and ≥50 as severe.^10^ All assessors classified participants as having either “clinical scabies” or “suspected scabies” or “not scabies” following the same criteria.

### Index test and reference standard.

After joint questioning about symptoms and contact history, the field-workers were instructed to examine the skin sequentially and without any discussion between them. The field-workers then recorded their results independently and blinded to one another into an electronic questionnaire using a mobile tablet device. The index test was the individual classification of each participant as clinical scabies, suspected scabies, or not scabies by the field-workers based on the skin assessment, combined with the participants’ response to the joint questioning.

The two medical doctors examined each participant independently and blinded to one another and entered their observations in the tablet. They then shared their results privately with another researcher (J. Furnival-Adams); if there were any disagreements, they discussed the case until they reached a consensus. The clinician’s joint assessment, based on the history of body itch at personal and household levels as well as their own clinical observations, was used as the reference standard. We have reported all aspects of the study recommended by STARD 2015 (Supplemental Table 1).

### Sample size.

We used the method proposed by Buderer, which allows estimation of the sample size required for clinically acceptable precision estimates of sensitivity and specificity, taking into consideration the prevalence of the disease in the study population^20^:

$$TP+FN=Z^{2}\frac{SN(1-SN)}{W^{2}}\;$$ (1)

$$N1=\frac{TP+FN}{P}$$ (2)

$$FP+TN=Z^{2}\frac{SP(1-SP)}{W^{2}}$$ (3)

$$N2=\frac{FP+TN}{\left(1-P\right)}\text{,}$$ (4)

where *TP* = true positives, *TN* = true negatives, *FN* = false negatives, *SN* = sensitivity, *SP* = specificity, *Z* = the standard normal, *P* = prevalence of the disease in the studied population, and *W* = the confidence estimates for the prevalence.

Equation (1) renders the number with a positive test, Equation (2) renders the sample size required for sensitivity, Equation (3) renders the number with a negative test, and Equation (4) renders the sample size required for specificity. The final sample size is the largest of *N*1 and *N*2.

For this study, we considered a sensitivity of 85% and a specificity of 74% as relevant, based on previous data.^13^ Assuming a prevalence estimate of 25% and a significance level of 0.05, a minimum of 192 participants were required for this assessment.

## STATISTICAL ANALYSES

For the scabies validation assessment, sensitivity, specificity, PPV, and NPV were calculated per field-worker for the diagnosis of scabies by comparing the index test to the reference standard. Cohen’s κ coefficients were calculated to assess interrater agreement between medical doctors and the rate of agreement between field-worker pairs.^21^ Pooled sensitivity, specificity, PPV, NPV, and κ estimates were calculated and reported in the main analysis.

Scores in the written training assessment were presented as a percentage. We assessed whether there were any associations between age, educational level, sex, and score using Pearson’s coefficient for age and a single-factor analysis of variance test for sex and educational level.

We used Excel (v. 1808, Microsoft, Redmond, WA) and RStudio, v. 4.1.1, (R Core Team [2022], R: A language and environment for statistical computing, R Foundation for Statistical Computing, Vienna, Austria; https://www.R-project.org/) to analyze the data.

## RESULTS

### Demographic characteristics of field-workers.

The median age of the nine field-workers was 23 years, and 78% were male (Table 1). All field-workers had completed grade 12 of high school.

**Table 1:** Demographic characteristics of field-workers

Variable	*n*	Median/%
Median Age, Years (IQR)	9	23 (21–28)
Sex		
Male	7	78%
Female	2	22%
Level of Education		
Basic*	0	0%
Medium	9	100%
Advanced	0	0%

IQR = interquartile range.

* Basic: completed grade 10 of high school, Medium: completed grade 12 of high school, and Advanced: completed a university degree.

### Training.

After training, the overall median assessment score was 85% (interquartile range [IQR] 81–90) (Supplemental Table 2). The field-workers performed best in the identification of scabies lesions. They had slightly lower scores in the scabies lesion count and the identification of infected scabies lesions. Although the scores were higher in all sections of assessment 2 than in assessment 1, the difference in scores was not statistically significant. There was no association between sex, age, or level of education and scores (Supplemental Table 3).

### Accuracy of field-workers’ assessments of scabies status.

In all, 193 participants were selected from the six clusters reporting the highest prevalence of scabies during visit 3 of the trial (1 month prior to the study). The median age of participants being examined for scabies was 16 years (IQR: 6–29 years), and 53% were female (Table 2). According to the reference standard, 26% of these participants met the diagnostic criteria for scabies; 55% of these cases were severe, 39% moderate, 6% mild, and 0% very mild. The majority (84%) of these cases were classified as clinical scabies. Each of the nine field-workers who took part in the study assessed varying numbers of participants with varying proportions of true scabies cases, as indicated in Table 3.

**Table 2:** Demographic characteristics and spectrum of presenting symptoms in study participants

Variable	*n*	Median/%
Median Age, Years (IQR)		16 (6–29)
Sex		
Male	90	47%
Female	103	53%
Total Scabies	51	26%
IACS Criteria Subcategory		
Clinical	43	22%
Suspected	8	4%
Not Scabies	142	74%
Severity of Scabies		
Very Mild		0
Mild	3	6%
Moderate	20	39%
Severe	28	55%

IACS = International Alliance for the Control of Scabies; IQR = interquartile range.

**Table 3:** Number of positive and negative scabies assessments per field-worker according to the reference standard and the field-worker

Field-Worker (index)	Participants Screened, *n*	Scabies (clinical or suspected), *n*		No Scabies, *n*	
		REF	FW	REF	FW
FW1	141	38	36	103	105
FW2	33	0	1	33	32
FW3	19	13	15	6	4
FW4	26	0	2	26	24
FW5	32	9	12	23	20
FW6	33	0	0	33	33
FW7	33	19	18	14	15
FW8	33	15	16	18	17
FW9	36	8	8	28	28

FW = field-worker; REF = reference standard.

Of the 193 participants, 47 of 51 true positive cases were correctly identified by both field-workers; however, on 12 occasions, true negative cases were mistakenly identified as positive, and on six occasions, true positives were falsely categorized as negative. The sensitivity of field-workers’ scabies assessment (considering both clinical and suspected cases) compared with medical doctor diagnosis was estimated to be 94.1% (95% CI: 89.7–98.7%), and the specificity was 95.8% (95% CI: 89.9–97.0%) (Table 4). When only clinical scabies cases were considered, the field-workers correctly identified 39 of the 43 true negative cases, but on nine occasions they misidentified clinical scabies cases as negative and on three occasions misclassified negative cases as positive. The estimated sensitivity of field-worker detection of clinical scabies was 89.5% (95% CI: 87.6–96.0%), and the specificity was 99% (95% CI: 97.9–100%). The estimated sensitivity of the field-workers’ detection of severe scabies cases was lower at 60.7% (95% CI: 47.9–73.5%), and the mean specificity was 97.1% (95% CI: 93.2–100%). Similar sensitivities were observed when considering moderate and mild cases; however, the specificity was lower for both moderate and mild scabies, suggesting a tendency for mild and moderate cases to be classified as severe. With respect to diagnosis of scabies (without consideration of severity or whether the case was clinical/suspected), the κ coefficient indicated perfect agreement between the medical doctors, with a value of 1.00. For the field-workers, the κ coefficient also indicated high levels of agreement, with a value of 0.95. Full 2 × 2 tables are available in Supplemental Table 4.

**Table 4:** Diagnostic accuracy of scabies diagnoses made by field-workers

Outcome	Mean Sensitivity (95% CIs)	Mean Specificity (95% CIs)	Mean PPV (95% CIs)	Mean NPV (95% CIs)	MD κ Coefficient	FW κ Coefficient
Scabies (clinical or suspected)	94.1 (89.7–98.7)	95.8 (89.9–97.0)	88.9 (83.1–94.9)	97.9 (94.0–99.3)	1.00	0.95
Clinical	89.5 (83.1–96.0)	99 (97.9–100)	96.3 (92.1–100)	97.1 (95.2–99.0)	0.96	0.97
Severe	60.7 (47.9–73.5)	97.1 (93.2–100)	94.0 (87.0–100)	76.2 (66.7–84.4)	0.84	0.53
Moderate	40.9 (23.4–52.8)	76.9 (69.8–88.1)	50 (32.7–67.3)	69.8 (60.1–79.5)	0.90	0.86
Mild/Very Mild	63.6 (32.7–67.3)	79.4 (60.1–79.5)	42.9 (23.4–52.8)	90.8 (69.8–88.1)	0.80	0.35

FW = Fieldworker; MD = Medical doctor; NPV = negative predictive value; PPV = positive predictive value.

## DISCUSSION

There is a significant gap in knowledge with respect to scabies prevalence in low- and middle-income countries, particularly in SSA. Our reliance on highly trained medical doctors and light microscopy for analysis of skin scraping samples means that it is often not feasible to conduct prevalence surveys. Existing research has indicated that nurses or other health professionals are capable of diagnosing scabies with a moderate-high level of accuracy. The results of this study suggest that individuals without any medical training may also be able to screen individuals for scabies with a similar degree of accuracy as medical doctors after a short, focal training. Given the global shortage of healthcare workers and costs associated with more specialized staff, this may provide a cost-effective alternative method of assessing community scabies prevalence.

When evaluating the accuracy of scabies (suspected or clinical) assessments in the field, our results suggested that diagnoses made by minimally trained field-workers compared with those by highly qualified medical doctors were highly sensitive and specific when considering diagnosis of scabies (either suspected or clinical). When considering the assessment of severity, field-worker assessments were less sensitive, and there was a tendency to misclassify milder cases as severe. There also appeared to be less agreement between medical doctors and between field-workers with respect to grading the severity of disease. The values of diagnostic accuracy were higher than expected and higher than observed in similar studies.^13^^–^^15^ This was most likely due to limitations in the choice of reference standard, as detailed below.

The main limitation of this study was that the reference standard used did not reflect the gold standard; therefore, the values were not representative of the true diagnostic accuracy. The gold standard for scabies diagnosis typically involves examination of the full body, often using dermoscopy for magnification of suspected lesions and burrows and microscopy of skin scrapings to confirm the presence of mites or mite products. In this study, however, medical doctors examined only exposed skin and did not take any samples for laboratory confirmation. Furthermore, because of the need for a translator in this study, the history of itch and contact history were shared between both field-workers and medical doctors. Although the reference standard used in our study is not perfect, it is similar to the methods typically used within the preventive medicine team in the study area. The proportion of severe cases in the study sample according to the reference standard is much higher than in other similar prevalence surveys, possibly suggesting that there were more mild cases of scabies that were not identified by the medical doctors.^13^^,^^22^ This may also be a factor contributing to the high diagnostic accuracy we observed.

## CONCLUSION

Despite the limitations of the study, the results do suggest that minimally trained workers with a secondary school education are capable of diagnosing scabies to a similar degree of accuracy as trained medical doctors. Given the paucity of scabies prevalence data at present and the limited resources available to support data collection in countries such as Mozambique, this may provide a valuable tool for rapid assessment of scabies prevalence where more specialized staff are not available and may facilitate the uptake of control measures. Mass drug administration programs used for parasitic diseases, including malaria, lymphatic filariasis, or scabies, may offer an opportunity for active case detection and monitoring of scabies. Further evaluations would be needed to better understand the true diagnostic accuracy of these assessments, how factors such as prevalence affect accuracy, and the contexts in which they are appropriate.

## Supplemental Materials

10.4269/ajtmh.24-0204 Supplemental Materials

## Data Availability

All data relevant to this article are publicly available in the dataverse of the University of Barcelona: https://dataverse.csuc.cat/dataset.xhtml?persistentId=doi:10.34810/data1100.
